# Sugammadex and Ideal Body Weight in Bariatric Surgery: The Debate Continues

**DOI:** 10.1155/2014/762432

**Published:** 2014-03-13

**Authors:** Michele Carron

**Affiliations:** Department of Medicine, Anesthesiology and Intensive Care, University of Padova, Via C. Battisti 267, 35121 Padova, Italy

I read with great interest the paper by Sanfilippo et al., evaluating the safety and efficacy of sugammadex administered on the basis of ideal body weight (IBW) to reverse neuromuscular blockade (NMB) at the reappearance of second twitch during train-of-four (TOF) stimulation [1]. However, this study raises several issues that deserve comment.

First, the authors chose a TOF ratio of 0.9 after sugammadex administration based on IBW as their primary endpoint [1]. A TOF ratio of 0.9 may not indicate full recovery, as this ratio can be associated with impaired neuromuscular transmission [2], inhibition of the hypoxic ventilatory response, and upper airway or pharyngeal dysfunction [3]. Based on acceleromyography studies, the current recommendation is that a TOF ratio greater than or equal to 1.0 should be used to confirm complete recovery from NMB [3].

Second, the TOF ratio observed before tracheal extubation was 0.90 ± 0.57 in the IBW group compared to 0.91 ± 0.08 observed in the real body weight (RBW) group [1]. Considering the standard deviation from the mean value, not all patients reached a TOF ratio greater than or equal to 0.9 before tracheal extubation in the IBW group [1]. In another study, a single dose of sugammadex based on IBW was associated with failure to achieve a TOF ratio greater than or equal to 0.9 in 23% and 40% of patients who had moderate and deep NMB, respectively [4]. These patients required a rescue dose of sugammadex, indicating that a high percentage of slow responders should be expected in obese patients and that there is potential risk of blockade reoccurrence [4].

Finally, the median time for recovery to a TOF ratio of 0.9 was slower in the IBW group than in the RBW group (151 ± 44 versus 121 ± 55 sec) [1]. Even if signs of residual curarization were not observed in the patients enrolled in the study [1], however, a slow recovery may be an expression of the ineffectiveness of the dose of sugammadex [5, 6] and the combination of underdosing and delayed response may be associated with recurarization [6]. So, the reversal of NMB with sugammadex dosed on the basis of IBW in obese patients requires a special observation in the postoperative period, being obese patients at risk of potential upper airway obstruction and respiratory failure [3–6]. Therefore, IBW does not appear to offer a proper dosing scalar for sugammadex administration in obese patients and requires close surveillance and neuromuscular monitoring during the postoperative period [3–6]. In contrast, with sugammadex administered on the basis of RBW, obtaining a TOF ratio greater than or equal to 1.0 with acceleromyography is a relatively easy goal to achieve [5–7].

Thus, the current evidence suggests that sugammadex administered to reverse NMB at the reappearance of second twitch during TOF stimulation on the basis of RBW has the full potential for improving care and safety of obese patients.
